# Evidence Based Weighing Policy during the First Week to Prevent Neonatal Hypernatremic Dehydration while Breastfeeding

**DOI:** 10.1371/journal.pone.0167313

**Published:** 2016-12-20

**Authors:** Suzanne Boer, Sevim Unal, Jacobus P. van Wouwe, Paula van Dommelen

**Affiliations:** 1 Department of Life Style, Netherlands Organization of Applied Scientific Research TNO, Leiden, the Netherlands; 2 Neonatal Intensive Care Unit, Ankara Children’s Hematology and Oncology Research Hospital, Ankara, Turkey; 3 Department of Child Health, Netherlands Organization of Applied Scientific Research TNO, Leiden, the Netherlands; Centre Hospitalier Universitaire Vaudois, FRANCE

## Abstract

**Background:**

Neonatal hypernatremic dehydration is prevented by daily neonatal weight monitoring. We aim to provide evidence-based support of this universally promoted weighing policy and to establish the most crucial days of weighing.

**Methods:**

Weight measurements of 2,359 healthy newborns and of 271 newborns with clinical hypernatremic dehydration were used within the first seven days of life to simulate various weighting policies to prevent hypernatremic dehydration; its sensitivity, specificity and positive predictive value (PPV) of these policies were calculated. Various referral criteria were also evaluated.

**Results:**

A policy of daily weighing with a cut-off value of -2.5 Standard Deviation Score (SDS) on the growth chart for weight loss, had a 97.6% sensitivity, 97.6% specificity and a PPV of 2.80%. Weighing at birth and only at days two, four and seven with the same -2.5 SDS cut-off, resulted in 97.3% sensitivity, 98.5% specificity and a PPV of 4.43%.

**Conclusion:**

A weighing policy with measurements restricted to birth and day two, four and seven applying the -2.5 SDS cut-off seems an optimal policy to detect hypernatremic dehydration. Therefore we recommend to preferably weigh newborns at least on day two (i.e. ~48h), four and seven, and refer them to clinical pediatric care if their weight loss increases below -2.5 SDS. We also suggest lactation support for the mother, full clinical assessment of the infant and weighing again the following day in all newborns reaching a weight loss below -2.0 SDS.

## Introduction

Breastfeeding is the most complete and balanced nutrition, it contains antibodies, enzymes, hormones and all the necessary nutrients in ideal proportions [1, 2]. Successful and exclusive breastfeeding has important benefits, to mothers, infants and society as a whole, both in the developed and developing countries: ‘it makes the world healthier, smarter and more equal’ [3]. However, in some cases successful initiation breastfeeding seems to fail due to inadequate latching, milk production or intake [4]. This may cause hypernatremic dehydration in the newborn [5]. The incidence of hypernatremic dehydration is estimated between 20 and 71 per 100.000 breast-fed infants, Among first time mothers incidences up to 223 per 100.000 are estimated [6–8]. Early excessive weight loss (> 9.3% around day 5) is its most obvious symptom [9], therefore, routine weight monitoring is universally proposed. Some studies imply that daily weighing, especially during the first five days, is the most effective intervention [10, 11]. Other studies state that weighing policies with less weight measurements are equally sufficient [5]. However, there is no evidence to support an effective choice. A reliable weighing policy detects at an early stage all cases with hypernatremic dehydration (high sensitivity) at the account of a very limited number of unnecessary referrals (high specificity). High specificity also prevents unnecessary parental concern causing early discontinuation of breastfeeding. Sensitivity and specificity however also relate to age; it is easier to find infants with hypernatremic dehydration at an older age [12]. Taking into account the severity of the condition, it is important to detect all cases at the onset of hypernatremic dehydration.

Recently we have developed reference charts for the weight loss in healthy breast-fed newborns that enables health care professionals to screen for hypernatremic dehydration during the first 10 days of life [13]. The Centiles or Standard Deviation Score (SDS) lines on this reference chart for weight loss may be used as a screening instrument: most cases with or who likely will develop hypernatremic dehydration fell below the -1 SDS line at day 3, the -2 SDS line at day 4, and the -2.5 SDS line at day 5 in this chart. In the present study, we aim to determine the best policy: what is the optimal number and exact timing of weighing moments during the first week of life by evaluating the sensitivities, specificities and positive predictive values (PPVs) of the various weighting policies practiced in neonatal and well-baby care.

The day of birth is defined as day zero. Weight loss in healthy breast-fed newborns occurs from day two on [13]. Mortality in newborns due to hypernatremic dehydration is reported from the fifth day of age and beyond. Permanent residual symptoms are reported in infants due to hypernatremic dehydration from the sixth day on [14,15]. Therefore a reliable weighing policy needs to include one or more weight measurements between day two and five after birth, and may include a measurement at the end of the first week to confirm that the infant is healthy and lactation successfully initiated. In order to develop evidence for a reliable weighing policy we aim to determine the optimal number and exact timing of weighing moments during the first week after delivery using available data from previous studies and the literature.

## Subjects and Methods

### Subjects

Longitudinal weights in the first 2 weeks of life were retrospectively obtained from healthy, exclusively breastfed newborns attending four primary care midwife practices from different parts of the Netherlands and from a literature search (S1 File, 102 cases,) as well as from patients admitted with hypernatremic dehydration at the Neonatal Intensive Care Unit, Ankara Children’s Hematology and Oncology Research Hospital, Ankara, Turkey. Hypernatremic dehydration is defined as a serum sodium concentration >149 mEq/L in otherwise asymptomatic neonates [14]. We obtained anonymous weight data from birth (= day 0). In the Netherlands, newborns are weighed at home by a midwife with a calibrated electronic scale. A midwife either assists the delivery at home or in an outpatient clinic, or is involved in the follow-up after hospital delivery by an obstetrician. The study design was in agreement with the Helsinki Declaration and approved by the Leiden University Medical Centre Medical Ethics Committee and the Ankara Children’s Hematology and Oncology Research Hospital Medical Ethics Committee. They did not require informed consent because it was a anonymous retrospect chart review. In total 2,359 birthweights of new-borns and 4,475 additional weight measurements between birth and postnatal day sixty were available (controls). In total, 271 cases of hypernatremic dehydration and their weight measurements up to postnatal age of day twenty-two were available for analyses. Since the presentation of hypernatremic dehydration is usually ranging from day three on [6], and none of our cases had been admitted at day one, we assume hypernatremic dehydration before day two to be nearly non-existent. In order to have accurate estimates at seven days of age, we selected data from all subjects within the first eight days.

### Methods

#### Simulation

Weight measurements were available from all subjects. Only part of the controls and none of the cases provided daily weight measurements up to eight days. We therefore generated a simulation by the following analysis in order to be able to obtain a daily sample of all controls and cases:

- First, weight loss in the sample of controls were standardized into SDS using the reference values for weight loss in healthy breast-fed newborns (n = 2,359) [13]. The SDS expresses the weight loss measurement relative to the reference population in units of standard deviation above or below the median. By definition, the SDS are standard normally distributed per day.
- Second, correlations of SDS between days were computed in the control sample. As part of the newborns in our sample had not been weighted daily, some of the correlations between different age intervals were not available and had to be interpolated from the known correlations. Therefore, each of the known correlations *r* was transformed using the Fisher’s transformation: *Z = 0*.*5 ln((1+r)/(1-r))*. Next, Table 1 shows the model fitted with the transformed correlation *Z* as outcome measure and a formula that depends on the age interval and the age of the first weight measurement [16]. The predicted Z-value were transformed back to correlation *r* with the formula: *r = (exp(2Z)-1/(exp(2Z)+1)*. This way, correlations between all different age intervals within the first eight days were obtained.
- Third, in order to obtain a sufficiently large sample (n = 10,000) of controls with daily weight measurements between the day of birth and postnatal day eight, a simulation was performed from a multivariate standard normal distribution with the correlations from the model in the second step in the covariance matrix.
- Fourth, in order to obtain a sample of 10,000 cases with hypernatremic dehydration and their daily weight measurements within the first eight days, we used the same simulation as performed in step three. The SDS of the cases were transformed to weight loss with the reference values of weight loss for cases. The weight loss was then converted back to SDS, with the reference values for weight loss in controls.

**Table 1:** Regression coefficients for the correlations between weight loss SDS in the first eight days of age (the variance accounted for 90.4%, the residual standard deviation is .453).

Item	Regression coefficient	Standard error	T-value on 20 d.f.*
Constant	0.6726	0.5639	1.193
ln(mean age)	1.2894	0.6466	1.994
ln(age interval)	-1.3140	0.4522	-2.906
(age interval)-1	-0.5362	0.4484	-1.196
ln(mean age) x ln(age interval)	0.2352	0.2236	1.052
ln2(mean age)	-0.2231	0.2639	-0.845

*Degrees of freedom

#### Analyses

The currently applied weighing policies in neonatal and well-baby care, weighing policies suggested in the literature and all combinations of weight measurements possible between postnatal day two up to seven days, were evaluated on sensitivity, specificity, and PPV. The evaluated policies differed not only in number and timing of weighing moments, but also on their cut-off values: the commonly used 10 percent rule of thumb for weight loss, as well as the recently proposed -2.0 or -2.5 SDS cut-offs on the reference chart for weight loss in healthy breast-fed newborns [13]. To validate our results, specificity of the optimum weighing policy was calculated based on the original data (those not simulated) of the controls. All data are in the Supporting Information (S2 and S3 Files). The reader may contact PVD (paula.vandommelen@tno.nl) for assistance when using these data.

## Results

Table 1 shows the regression coefficients for the model to obtain the correlations between weight loss on all first eight days. The model accounted for 90.4 percent of the variation. The obtained correlations of the SDS are presented in Table 2. The smaller the age interval between the consecutive days and the higher the age at the first measurement, the stronger the correlations. These correlations were used in the multivariate standard normal distribution in order to simulate the daily measurements for the controls and the cases with hypernatremic dehydration.

**Table 2:** Correlations between weight loss SDS for the first eight days (N).

	**Day 1**	**Day 2**	**Day 3**	**Day 4**	**Day 5**	**Day 6**	**Day 7**	**Day 8**
**Day 1**	-							
**Day 2**	.553	-						
**Day 3**	.374	.811	-					
**Day 4**	.274	.675	.886	-				
**Day 5**	.219	.564	.793	.917	-			
**Day 6**	.188	.484	.703	.849	.934	-		
**Day 7**	.170	.426	.629	.778	.881	.943	-	
**Day 8**	.161	.384	.569	.714	.823	.901	.950	-

While investigating all different weighing policies, small differences were noticed in sensitivity and specificity for weighing policies that differ in number of weight measurements. In contrast, sensitivity and specificity depended highly on the timing of weight measurements. Sensitivity and specificity strongly improved in weighing policies that include high ages (e.g. postnatal day seven). Specificity and PPV were considerably lower for the -2.0 SDS cut-off on the growth chart for weight loss and the 10 percent rule of thumb, than for the -2.5 SDS cut-off.

Daily weighing with the -2.5 SDS cut-off had a sensitivity of 97.6 percent, a specificity of 97.6 percent, and a PPV of 2.80 percent (Table 3). This table also shows various weighing policies with a first weight measurement at the second day of age and a last weight measurement at the seventh day. Sensitivity and specificity differ slightly, independent to the number of weight measurements within the weighing policy. Weighing at birth and days two, four and seven and applying the -2.5 SDS cut-off showed the highest PPV (4,43), and had a sensitivity >97% and a specificity >98%.

**Table 3:** Statistical values of different weighing policies (N).

Policy Day	Sensitivity	Specificity	PPV	Cut-off
2, 3, 7	97.3	98.5	4.30	-2.5 SDS
2, 3, 7	98.8	94.8	1.32	-2.0 SDS
2, 3, 7	96.4	93.8	1.09	10% rule
2, 4, 7	97.3	98.5	4.43	-2.5 SDS
2, 4, 7	98.8	94.9	1.36	-2.0 SDS
2, 4, 7	97.6	94.7	1.28	10% rule
2, 5, 7	97.3	98.5	4.38	-2.5 SDS
2, 3, 4, 7	97.4	98.3	4.00	-2.5 SDS
2, 3, 5, 7	97.4	98.3	3.96	-2.5 SDS
2, 3, 6, 7	97.5	98.3	3.81	-2.5 SDS
2, 4, 5, 7	97.4	98.4	4.15	-2.5 SDS
2, 4, 6, 7	97.5	98.3	3.96	-2.5 SDS
2, 3, 4, 5, 6, 7	97.6	97.6	2.80	-2.5 SDS

## Discussion

**Fig 1:**
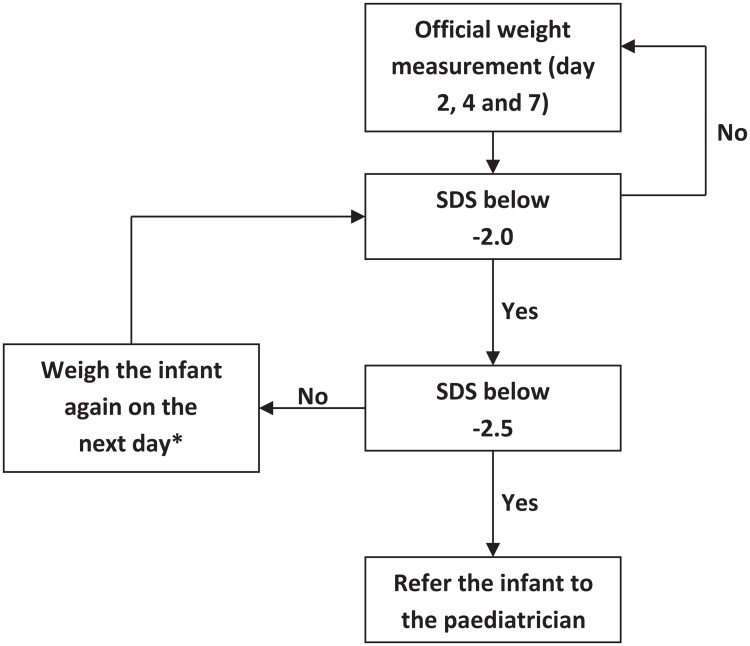
Flowchart for the evidence based weighing policy to prevent neonatal dehydration. * Other clinical symptoms: feeding patterns, number of wet diapers, frequency and quality of stools, evaluation of pattern of breastfeeding and intervention if necessary. Monitor the infant when mild clinical symptoms are present and refer the infant to the paediatrician when severe clinical symptoms are present.

In the present study, we found that the optimum weighing policy consists of weight measurements at birth and on the second, fourth and seventh day of age, applying the -2.5 SDS cut-off on the reference chart for weight loss in the healthy breast-fed controls. As far as we know, our study is the first to develop evidence to support such a weighing policy. Our weighing policy is represented in Fig 1. There is no need to weigh newborns at the first day, after their birthweight was taken at day zero. Literature shows none of the newborns suffer from hypernatremic dehydration before day two. This supports the choice for the first weight measurement at day two. It is important to early detect newborns with hypernatremic dehydration as permanent residual symptoms and even mortality in newborns with hypernatremic dehydration do occur (from the fifth day of age and beyond) [14,15].

A second weight measurement was added to the fourth day of age, due to higher diagnostic validity than a weighing policy with a second weight measurement on the third day of age. To confirm that the newborn is healthy, a third weight measurement was added at the end of the first week, at day seven postnatal age. This optimum weighing policy with weight measurement at birth and the second, fourth and seventh day of age, resulted in a specificity of 99.1 percent in the original, not simulated, sample of healthy breast-fed newborns. Besides clinical diagnostics, other important considerations had to be made in order to choose the most valid weighing policy. Not only the severity of the condition had to be taken into account, also the minimum age of referral, as well as the prevalence of hypernatremic dehydration, and the sensitivity and specificity of the instrument. The last three aspects were also represented by the PPV. Eventually, the final choice for the optimum weighing policy was made taking into account all of the above aspects.

Weighing is considered to be important in the assessment of newborns’ growth and hydration status. Weight change provides the most objective and clear criterion for referral, but an abnormal early weight loss is not the only symptom of hypernatremic dehydration. According the studies of Boskabadi [9] and Pelleboer [7] the main reasons for admission were fever, lethargy, jaundice, irritability, seizure and just a minority had excessive weight loss of more than 10.0 percent as the main reason for admission. With those findings we want to emphasize the need for a proper weighing policy, but also focus on other clinical symptoms, even when weight loss is still within the normal range. This weighing policy can contribute to the early detection of hypernatremic dehydration in newborns.

In our study, the majority of cases had an observed age of detection beyond the range of days within our weighing policy [14, 15]. However, we assumed that all hypernatremic dehydration newborns follow the growth pattern of the reference chart for newborns with hypernatremic dehydration in the first seven days. Therefore, we expect that newborns with a later age of detection, have similar growth patterns as newborns with an early age of detection. This seems a valid assumption, since it is very unlikely for a case to instantly lose an abnormal amount of weight when detected late. Furthermore, we assumed that all newborns with hypernatremic dehydration were already a case at day three being the earliest range quoted in the literature [6]. Reasons for the late age of detection could be a lack of knowledge about hypernatremic dehydration and the associated symptoms, or an insufficient or no weighing policy.

Special attention is needed for newborns with an increased risk of developing hypernatremic dehydration. The risk of developing hypernatremic dehydration is three fold higher for newborns born by a caesarean section than for those who born vaginally [10]. Other risk factors include prim parity, delay of initiation of first breastfeeding, newborn’s hunger cues are slight of non-existent, use of the nipple shield and infrequent feeding [8, 9].

A limitation of our study is that the correlations of weight loss SDS between days were calculated based on healthy breast-fed controls that were not daily measured. Moreover, we assumed that the correlations would be the same for cases with hypernatremic dehydration, since no longitudinal data on weight loss were available for newborns with hypernatremic dehydration. This seems a valid assumption, since the correlations between birth weight and weight at day three up to day eight were almost similar between the controls and cases with hypernatremic dehydration. Another limitation is that the growth chart for cases with hypernatremic dehydration was based on a relatively small sample of 271 newborns, however the number is relatively large in relation to its prevalence. Therefore, the estimated centiles could be less precise, especially for the more extreme centiles ±2.0 SDS and ±2.5 SDS. However, due to large differences between the reference charts at an early age, the extreme centiles become less important. A strength of our study is that we used a very large sample of healthy breast-fed newborns as controls. Also, we have used quite advanced statistical methods to provide this evidence on the various weighing policies.

We conclude that a weighing policy with weight measurements at birth and the second, fourth and seventh day, can contribute to an early detection and likely prevention of newborns with hypernatremic dehydration at the account of only a small number of unnecessary referrals to the paediatrician. Newborns with a weight loss below -2.5 SDS need to be referred, and newborns with a weight loss below -2.0 SDS need further attention, such as more intensive lactation support for the mother [17], full clinical assessment and an additional weight measurement of the infant the following day. We recommend further research to validate our findings in new prospective studies that actually apply this policy with a sufficient size sample of subjects, in combination with data on the occurrence of other specific pediatric symptoms or obstetric factors, like induction of labor and amounts on iv fluids received intrapartum [18].

## Supporting Information

S1 File References literature search. (DOCX)

S2 File Data healthy subjects. (SAV)

S3 File Data cases. (SAV)
