# Supplementary material for: Evidence Based Weighing Policy during the First Week to Prevent Neonatal Hypernatremic Dehydration while Breastfeeding
Source: PLoS One. 2016 Dec 20;11(12):e0167313. doi: 10.1371/journal.pone.0167313 (PMC5172525; doi:10.1371/journal.pone.0167313)
Supplement: S1 File — (DOCX) [file pone.0167313.s001.docx]

**Supplementary file: list of manuscripts included in out literature search**

1 (2001) The Optimal Duration of Exclusive Breastfeeding; Report of an Expert Consultation. Cited 606 times. Genève: WHO

2 Lanting, C.I., Herschderfer, K., Van Wouwe, J.P., Reijneveld, S.A. (2003) Effect van de Invoering van het 'Baby Friendly Hospital Initiative' op het Geven van Borstvoeding in Nederland Publicatienr 03.212. Leiden: TNO Preventie en Gezondheid

3 Clarke, T.A., Markarian, M., Griswold, W., Mendoza, S. Hypernatremic dehydration resulting from inadequate breast-feeding (1979) Pediatrics, 63 (6), pp. 931-932

4 Laing, I.A., Wong, C.M. Hypernatraemia in the first few days: Is the incidence rising? (2002) Archives of Disease in Childhood: Fetal and Neonatal Edition, 87 (3), pp. F158-F162

5 Oddie, S., Richmond, S., Coulthard, M. Hypernatraemic dehydration and breast feeding: A population study (2001) Archives of Disease in Childhood, 85 (4), pp. 318-320. doi: 10.1136/adc.85.4.318

6 Acharya, P.T., Payne, W.W. Blood chemistry of normal full-term infants in the first 48 hours of life (1965) Archives of Disease in Childhood, 40 (212), pp. 430-435. doi: 10.1136/adc.40.212.430

7 Amitai, I., Goder, K., Husseini, N., Rousso, M. Hypernatremic dehydration complicated by peripheral gangrene in infancy (1983) Israel Journal of Medical Sciences, 19 (6), pp. 538-540

8 Anand, S.K., Sandborg, C., Robinson, R.G., Lieberman, E. Neonatal hypernatremia associated with elevated sodium concentration of breast milk (1980) The Journal of Pediatrics, 96 (1), pp. 66-68. doi: 10.1016/S0022-3476(80)80330-1

9 Arboit, J.M., Gildengers, E. Breast-feeding and hypernatremia (1980) The Journal of Pediatrics, 97 (2), pp. 335-336 doi: 10.1016/S0022-3476(80)80522-1

10 Bajpai, A. Hypernatremic dehydration in a neonate. (2002) Indian Pediatrics, 39 (6), pp. 599-600; discussion 601

11 Becker, P.G., Conard, J.A. Breast-feeding failure. (1990) Indiana medicine : the journal of the Indiana State Medical Association, 83 (9), pp. 648-650

12 Swarna, R.B., Lewis, P., Dinakar, C. Hypernatremic dehydration in a neonate (2001) Indian Pediatrics, 38 (10), pp. 1174-1177

13 Boumahni, B., Pyaraly, S., Randrianaly, H., Robillard, P.Y., Renouil, M. Hypernatremic dehydration and breast feeding (2001) Archives de Pediatrie, 8 (7), pp. 731-733. doi: 10.1016/S0929-693X(00)00307-9

14 Canet, E., Canet, E., Perrain, M.F. Quel est votre diagnostic? (1984) Concours Med, 106, pp. 1689-1691

15 Chilton, L.A. Prevention and management of hypernatremic dehydration in breast-fed infants (1995) Western Journal of Medicine, 163 (1), pp. 74-76

16 Cooper, W.O., Atherton, H.D., Kahana, M., Kotagal, U.R. Increased incidence of severe breastfeeding malnutrition and hypernatremia in a metropolitan area (1995) Pediatrics, 96 (5 I), pp. 957-960

17 Ellis, D., Kaye, R.D., Bontempo, F.A. Aortic and renal artery thrombosis in a neonate: Recovery with thrombolytic therapy (1997) Pediatric Nephrology, 11 (5), pp. 641-644. doi: 10.1007/s004670050356

18 Ernst, J.A., Wynn, R.J., Schreiner, R.L. Starvation with hypernatremic dehydration in two breast-fed infants. (1981) Journal of the American Dietetic Association, 79 (2), pp. 126-130

19 Gebara, B.M., Everett, K.O. Dural sinus thrombosis complicating hypernatremic dehydration in a breastfed neonate (2001) Clinical Pediatrics, 40 (1), pp. 45-48

20 Ghishan, F.K., Roloff, J.S. Malnutrition and Hypernatremic Dehydration in Two Breast-fed Infants (1983) Clinical Pediatrics, 22 (8), pp. 592-594 doi: 10.1177/000992288302200818

21 Gilmore, H.E., Rowland, T.W. Critical Malnutrition in Breast-Fed Infants: Three Case Reports (1978) American Journal of Diseases of Children, 132 (9), pp. 885-887 doi: 10.1001/archpedi.1978.02120340061011

22 Harding, D., Moxham, J., Cairns, P. Weighing alone will not prevent hypernatraemic dehydration [1] (2003) Archives of Disease in Childhood: Fetal and Neonatal Edition, 88 (4), p. F349

23 Hatzidaki, E., Manoura, A., Korakaki, E., Mamoulakis, D., Kokori, H., Giannakopoulou, C. Breast feeding - When nature fails to satisfy (2001) Clinical and Experimental Obstetrics and Gynecology, 28 (4), pp. 253-254

24 Heldrich, F.J., Shaw, S.S. Case report and review of literature: hypernatremia in breast-fed infants. (1990) Maryland medical journal (Baltimore, Md. : 1985), 39 (5), pp. 475-478

25 Hilliard, T.N., Marsh, M.J., Malcolm, P., Murdoch, I.A., Wood, B.P. Sagittal sinus thrombosis in hypernatremic dehydration (1998) Arch Pediatr Adolesc Med, 152, p. 1147

26 Jaffe, K.M., Kraemer, M.J., Robison, M.C. Hypernatremia in breast-fed newborns.

(1981) Western Journal of Medicine, 135 (1), pp. 54-55

27 Jaramillo, I., Lopez, G., Hernandez, H. Hypernatremic dehydration and death in an infant [2] (2003) Pediatric Emergency Care, 19 (1), pp. 62-63

28 Kaplan, J.A., Siegler, R.W., Schmunk, G.A. Fatal hypernatremic dehydration in exclusively breast-fed newborn infants due to maternal lactation failure (1998) American Journal of Forensic Medicine and Pathology, 19 (1), pp. 19-22 doi: 10.1097/00000433-199803000-00003

29 Kini, N., Zahn, S., Werlin, S.L. Hypernatremic dehydration in breast-fed infants (1995) Wisconsin Medical Journal, 94 (3), pp. 143-145

30 Korkmaz, A., Yiǧit, S., Firat, M., Oran, O. Cranial MRI in neonatal hypernatraemic dehydration (2000) Pediatric Radiology, 30 (5), pp. 323-325 doi: 10.1007/s002470050749

31 Livingstone, V.H., Willis, C.E., Abdel-Wareth, L.O., Thiessen, P., Lockitch, G. Neonatal hypernatremic dehydration associated with breast-feeding malnutrition: A retrospective survey (2000) CMAJ, 162 (5), pp. 647-652

32 Macdonald, P.D., Ross, S.R.M., Grant, L., Young, D. Neonatal weight loss in breast and formula fed infants (2003) Archives of Disease in Childhood: Fetal and Neonatal Edition, 88 (6), pp. F472-F476

33 Marino, R., Gourji, S., Rosenfeld, W. Neonatal metabolic casebook. Hypernatremia and breast feeding. (1989) Journal of perinatology : official journal of the California Perinatal ssociation, 9 (4), pp. 451-453

34 Mercier, J.C., Outin, S., Paradis, K. Hypernatremic dehydration and breast-feeding (1986) Archives Francaises de Pediatrie, 43 (7), pp. 465-470

35 Molteni, K.H. Initial Management of Hypernatremic Dehydration in the Breastfed Infant (1994) Clinical Pediatrics, 33 (12), pp. 731-740 doi: 10.1177/000992289403301205

36 Ng, P.C., Chan, H.B., Fok, T.F., Lee, C.H., Chan, K.M., Wong, W., Cheung, K.L. Early onset of hypernatraemic dehydration and fever in exclusively breast-fed infants (1999) Journal of Paediatrics and Child Health, 35 (6), pp. 585-587 doi: 10.1046/j.1440-1754.1999.00428.x

37 Niestijl, A.L., Sauer, P.J.J. Breast-feeding sometimes insufficient during the first few days after birth (2003) Nederlands Tijdschrift voor Geneeskunde, 147 (49), pp. 2405-2407

38 Pascale, J.A., Brittian, L., Lenfestey, C.C., Jarrett-Pulliam, C. Breastfeeding, dehydration, and shorter maternity stays. (1996) Neonatal network : NN, 15 (7), pp. 37-43

39 Paul, A.C., Ranjini, K., Muthulakshmi, Roy, A., Kirubakaran, C. Malnutrition and hypernatraemia in breastfed babies (2000) Annals of Tropical Paediatrics, 20 (3), pp. 179-183

40 Peters, J.M Hypernatremia in breast-fed infants due to elevated breast milk sodium (1989) Journal of the American Osteopathic Association, 89 (9), pp. 1165-1170

41 Rand, S.E., Kolberg, A. Neonatal hypernatremic dehydration secondary to lactation failure (2001) Journal of the American Board of Family Practice, 14 (2), pp. 155-158

42 Roddey, O.F., Martin, E.S., Swetenburg, R.L. Critical Weight Loss and Malnutrition in Breast-fed Infants: Four Case Reports (1981) American Journal of Diseases of Children, 135 (7), pp. 597-599 doi: 10.1001/archpedi.1981.02130310003002

43 Rosenfeld, W., Lopez de Romana, G., Kleinman, R., Finberg, L. Clinical Review: Improving the Clinical Management of Hypernatremic Dehydration: Observations from a Study of 67 Infants with This Disorder (1977) Clinical Pediatrics, 16 (5), pp. 411-417 doi: 10.1177/000992287701600501

44 Rowland, T.W., Zori, R.T., Lafleur, W.R., Reiter, E.O. Malnutrition and Hypernatremic Dehydration in Breast-Fed Infants (1982) JAMA: The Journal of the American Medical Association, 247 (7), pp. 1016-1017 doi: 10.1001/jama.1982.03320320052031

45 Rushton, A.R., Lambert, G.P., Katcher, A.L., Frangakis, D. Dehydration in a Breast-Fed Infant (1982) JAMA: The Journal of the American Medical Association, 248 (6), p. 646 doi: 10.1001/jama.1982.03330060014010

46 Scott, J.X., Raghunath, Gnananayagam, J.E.J., Simon, A. Neonatal hypernatraemic dehydration and malnutrition associated with inadequate breastfeeding and elevated breast milk sodium (2003) Journal of the Indian Medical Association, 101 (5), pp. 318-321

47 Sofer, S., Ben-Ezer, D., Dagan, R. Early severe dehydration in young breast-fed newborn infants (1993) Israel Journal of Medical Sciences, 29 (2-3), pp. 85-89

48 Thullen, J.D. Management of Hypernatremic Dehydration due to Insufficient Lactation (1988) Clinical Pediatrics, 27 (8), pp. 370-372 doi: 10.1177/000992288802700803

49 Van Amerongen, R.H., Moretta, A.C., Gaeta, T.J. Severe hypernatremic dehydration and death in a breast-fed infant (2001) Pediatric Emergency Care, 17 (3), pp. 175-180 doi: 10.1097/00006565-200106000-00006

50 Van der Heide, P.A., Toet, M.C., Van Diemen-Steenvoorde, J.A.A.M., De Lavalette, P.A.W.A.R., De Jonge, G.A. Hypertonic dehydration in 'silent' malnutrition of breast-fed infants (1998) Nederlands Tijdschrift voor Geneeskunde, 142 (18), pp. 993-995

51 Willis, C.E., Livingstone, V. Infant Insufficient Milk Syndrome Associated with Maternal Postpartum Hemorrhage (1995) Journal of Human Lactation, 11 (2), pp. 123-126 doi: 10.1177/089033449501100218

52 Macdonald, P.D., Grant, L., Ross, S.R.M. Hypernatraemia in the first few days: A tragic case [3] (2003) Archives of Disease in Childhood: Fetal and Neonatal Edition, 88 (4), p. F350
